# Nasal Placode Development, GnRH Neuronal Migration and Kallmann Syndrome

**DOI:** 10.3389/fcell.2019.00121

**Published:** 2019-07-11

**Authors:** Hyun-Ju Cho, Yufei Shan, Niteace C. Whittington, Susan Wray

**Affiliations:** Cellular and Developmental Neurobiology Section, National Institute of Neurological Disorders and Stroke, National Institutes of Health, Bethesda, MD, United States

**Keywords:** GnRH, neurodevelopment, olfactory placode, neuronal migration, olfactory system, Kallmann syndrome

## Abstract

The development of Gonadotropin releasing hormone-1 (GnRH) neurons is important for a functional reproduction system in vertebrates. Disruption of GnRH results in hypogonadism and if accompanied by anosmia is termed Kallmann Syndrome (KS). From their origin in the nasal placode, GnRH neurons migrate along the olfactory-derived vomeronasal axons to the nasal forebrain junction and then turn caudally into the developing forebrain. Although research on the origin of GnRH neurons, their migration and genes associated with KS has identified multiple factors that influence development of this system, several aspects still remain unclear. This review discusses development of the olfactory system, factors that regulate GnRH neuron formation and development of the olfactory system, migration of the GnRH neurons from the nose into the brain, and mutations in humans with KS that result from disruption of normal GnRH/olfactory systems development.

## Introduction

**FIGURE 1:**
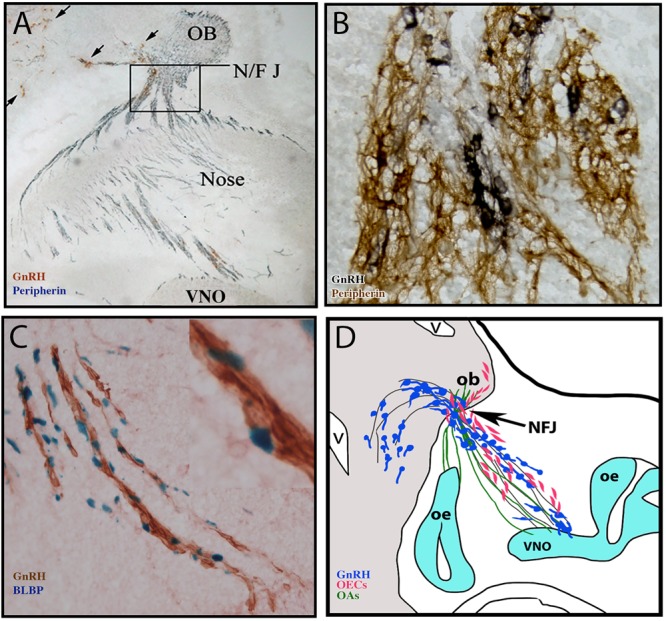
Multiple components form the GnRH migratory bundle during development. **(A)** Immunocytochemistry staining of E14.5 mouse section. Brown, GnRH (black arrows); Blue, peripherin (marking olfactory axons). Axons and cells must travel through the cribriform plate at the NFJ (boxed area) **(B)** Immunocytochemistry staining of GnRH (black) and peripherin (brown) at the nasal forebrain junction (NF/J). **(C)** Staining of GnRH neurons (brown) and BLBP (blue nuclei, labeling OECs) on the migratory route. **(D)** Schematic of multiple components forming the GnRH migratory bundle in mouse at E14.5. GnRH neurons (blue) migrate along VNO/terminal nerve axons (black lines) into the forebrain. Other VNO sensory axons bundle with olfactory sensory axons (green) and OECs (pink) enter the olfactory bulb.

Unraveling how specialized neurons arise from heterogenous cell populations and then migrate to their appropriate location has important implications for understanding the development and progression of neuronal disorders. Proper establishment of the Gonadotropin releasing hormone-1 (GnRH) system is crucial for function of the reproduction system in vertebrates. GnRH neurons originate within the nasal placode (Schwanzel-Fukuda and Pfaff, 1989; Wray et al., 1989a,b), a region that also gives rise to olfactory sensory neurons (both those that sense odors as well as those that sense pheromones) and olfactory ensheathing cells (OECs). As the nasal placode invaginates to form the main olfactory epithelium (OE) and the vomeronasal organ (VNO), GnRH neurons migrate out of the VNO to the brain along axons that are covered by OECs (Figure 1; Wray, 2002a). Once within the forebrain, GnRH neurons function in hormone signaling through the hypothalamic-pituitary-gonadal (HPG) axis. In humans, improper development of the nasal placode and/or migration of GnRH neurons results in various forms of hypogonadism, including Kallmann Syndrome (KS) which is characterized by anosmia and lack of sexual development. Ongoing studies continue to support the occurrence of oligogenism in KS patients, indicating that a combination of mutations or rare variants on two or more genes can underlie the disease (di/oligogenic disorder). As such, one needs to understand the cellular components that GnRH neurons are exposed to, how they interact, which are redundant and who may compensate. In addition, the question remains as to the diversity of GnRH cells themselves – Do subpopulations exist (expressing different receptors perhaps), responding to different cues, to ensure some GnRH cells reach the brain and the animal can reproduce? Since the development of the GnRH system is intimately entwined with the development of the olfactory system, factors that regulate development of the nasal placode and thus GnRH cells are discussed in Section “Development of the Olfactory Placode and GnRH Neurons”. Section “Migration of GnRH Neurons From the Nose to the Brain” focuses on factors that influence outgrowth of olfactory axons and migration of the GnRH neurons. Then, in the last section, we discuss mutations in humans with KS that result from disruption of normal development of the GnRH/olfactory systems and present new candidate genes that might contribute to KS based on bioinformatic analysis of known KS genes.

## Development of the Olfactory Placode and GnRh Neurons

**FIGURE 2:**
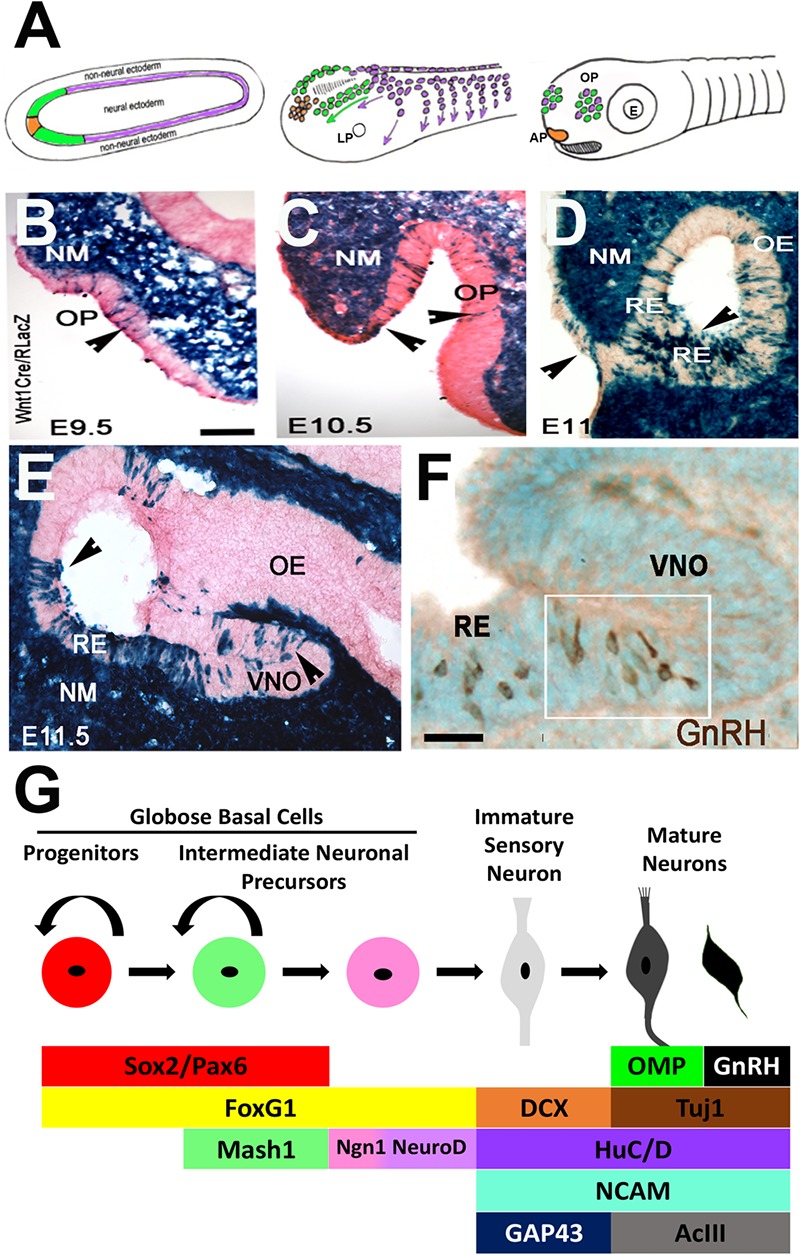
Development of the olfactory system. **(A)** Schematic of olfactory placode formation. Cell populations at the border of the neural plate: preplacodal ectoderm (green), anterior pituitary placode (orange), and pre-migratory neural crest cells (purple), migrate rostrally and intermix to form the olfactory placodes. **(B–E)** X-Gal staining on nasal sections of E9.5–E11.5 neural crest reporter mouse (Wnt1Cre/RLacZ) showing Wnt1 expression (blue) in cells within the invaginating olfactory pit (OP, arrowheads), olfactory epithelium (OE), vomeronasal organ (VNO) and throughout the nasal mesenchyme (NM) and respiratory epithelium (RE). **(F)** Immunocytochemistry staining of E11.5 OE showing GnRH neurons (brown) located at the border between the RE and developing VNO. **(G)** Schematic of olfactory neurogenesis. Cell types in the OE can be identified by expression of specific factors, which direct cells to remain as cycling progenitors or to undergo neuronal differentiation to form sensory and/or GnRH neurons (Adapted from Whitlock et al., 2006; Forni et al., 2011).

Olfactory placodes (OP) are complex structures forming as bilateral transient thickenings of non-neural ectoderm at the ventrorostral region of the developing embryonic head (Figure 2A). They arise from pre-placodal ectoderm (reviewed in Saint-Jeannet and Moody, 2014) and intermixing of migratory cranial neural crest cells and ectodermal cells occur in the OP in vertebrates (Baker and Bronner-Fraser, 2001; Whitlock, 2005b; Forni et al., 2011). These neural crest cells give rise to the OECs (Forni et al., 2011; Katoh et al., 2011). However, the contribution of neural crest to other cell types in the OP is still being debated. Transgenic reporter mice were used to map the development of ectoderm and neural crest cells arising in the OE and VNO, and neural crest cells were found to give rise to a subpopulation of progenitors, sensory neurons, support cells and GnRH cells (Forni et al., 2011) (Figure 2A).

In mice, the OP forms as an epithelial sheet at embryonic day (E) 9.5 (Figure 2B–F). During the next 24 h, this sheet of cells invaginates to form the olfactory pit, which is the beginning of the nasal cavity and comprised of OE and surrounding nasal mesenchyme. At E11.5 the pit deepens to form the nostrils. The olfactory pit contains neural and non-neural structures and forms sensory and respiratory epithelium. The respiratory epithelium is located at the rostral portion and is the source of FGF8 and BMP4 expression that define the borders of the neurogenic sensory epithelium niche (Forni et al., 2013). The sensory epithelium, located within the invaginating OP, is layered and contains stem and progenitor cells, sensory neurons, GnRH neurons, sustentacular support cells and mucus-producing Bowman’s glands. Early on, OECs appear to move to the lamina propria beneath the basal membrane where they start to surround olfactory axons. Following invagination, GnRH neurons delaminate from the OE and migrate with immature neurons (Fornaro et al., 2003) and OECs to the brain along axonal projections. As development progresses, the OE forms into the VNO and turbinates of the main olfactory epithelium (mOE). The VNO is formed medially in the ventral region of the OE and gives rise to pheromone receptor cells that project to the accessory olfactory bulb, whereas the mOE generates odor receptors that project to the main olfactory bulb. Disruption of several of these sequences have consequences on GnRH neuronal specification and migration and thus reproductive capacity of the animal.

Cell stages can be identified by the expression of specific transcription factors and histological arrangement within the OE. Progenitor cells are first located on both the apical and basal sides of the OE, but later become restricted to the basal side and are identified as horizontal and globose basal cells. Globose basal cells are actively dividing progenitor cells that give rise to neurons (Figure 2G), while horizontal basal cells are stem cells that give rise neuronal and non-neuronal cells in the OE (Schwob et al., 2017). Multiple transcriptions factors have been identified as important for this process. PAX6 is required for initial establishment of placodal fate. Both PAX6 and SOX2 regulate the progression of olfactory neurogenesis (Donner et al., 2007; Tucker et al., 2010; Moody and LaMantia, 2015), holding olfactory progenitors in an undifferentiated state and preventing OSN formation (Packard et al., 2016). PAX6-null mice do not develop an eye or nasal placode, and thus lack olfactory cells and GnRH neurons (Dellovade et al., 1998). FOXG1 is only expressed in ventral progenitor cells, yet it plays a global role in OE formation. The loss of FOXG1 results in a diminished OE without neuronal differentiation (Duggan et al., 2008), and a loss of proliferation and/or differentiation (Kawauchi et al., 2009). Analysis of GnRH neurons and OECs was not examined. MASH1 (or ASCL1) is a basic helix-loop-helix (bHLH) transcription factor that is expressed in intermediate neuronal precursors (Figure 2G) that will give rise to OSNs, both in the OE and VNO. MASH1 is essential for proper olfactory neuron development (Cau et al., 1997; Castro et al., 2011), activating Notch signaling in the OE (Cau et al., 2002), which is required for differentiation. Sensory neurons of the VNO are also dependent on MASH1 for their proper development, with the size of the accessory olfactory bulb reduced when MASH1 is knocked out (Murray et al., 2003). While MASH1 regulates olfactory neurogenesis, it does not regulate OP formation or OE development, as a deletion of MASH1 results in an OE that still expresses SOX2 and has no defects in OE structure (Kawauchi et al., 2004). MASH1 activates a number of downstream targets, including Neurogenin-1, another bHLH proneural transcription factor. Neurogenin-1 marks basal cells (Ma et al., 1997) that give rise to daughter cells that exit the cell cycle to become immature OSNs and activates expression of NEUROD1, which is expressed in post-mitotic immature OSNs (Cau et al., 1997). Once intermediate progenitor cells commit to the olfactory neuronal lineage and differentiate, they become bipolar and start migrating away from the basal layer, and express markers such as NCAM (Calof and Chikaraishi, 1989), microtubule protein Doublecortin (DCX) (Francis et al., 1999; Gleeson et al., 1999), and HuC/D (Okano and Darnell, 1997; Fornaro et al., 2001) and GAP-43 (Pellier et al., 1994). As they mature, OSNs extend their cilia into the nasal cavity on the apical surface for odor detection, and project their OEC-wrapped axons to the olfactory bulb (Kawauchi et al., 2009). Molecules such as TUJ1 (De Carlos et al., 1996), adenylyl cyclase (ACIII) (Wong et al., 2000) and Olfactory Marker Protein (OMP) (Margolis, 1972) are expressed in mature OSNs (Figure 2G). In neurogenin-1 mutant OE, most OSNs fail to differentiate even though basal progenitors are still generated (Cau et al., 2002). Notably, GnRH cells do not appear to arise from MASH1 positive progenitors (Kramer and Wray, 2000a), suggesting differences among intermediate neuronal precursors in the VNO.

Thirty years after the identification of the nasal placode as the source of forebrain GnRH cells, the lineage and specification of GnRH neurons still remains unclear. Lineage tracing and ablation studies in mouse have shown that GnRH cells in the OE arise from two precursor populations having ectodermal and neural crest lineages (reviewed in Wray, 2002a; Forni and Wray, 2012). The formation of GnRH neurons has also been analyzed using an activating enhancer binding protein 2 (AP2) alpha mutant mouse. AP2alpha is expressed in respiratory epithelium. In these mutants, GnRH neurons were ectopically expressed although the morphology of the OE and VNO was conserved (Kramer et al., 2000). Unfortunately, embryonic stages earlier than E12.5 were not examined, but these data suggest alternative regulation of GnRH and OSN development and add to the argument that lineage and specification of GnRH neurons and sensory neurons diverge early. Identifying a marker that distinguishes GnRH cell lineage has been difficult. In mice, GnRH cells mature at approximately E11.5 and begin migrating out of the OE toward the forebrain. It was hypothesized that two bilateral groups of 50 progenitors (100 in total) give rise to a population of about 800 GnRH cells at E12.5 (Wray et al., 1989a). Nestin was found to be expressed in primary GnRH cells prior to onset of GnRH mRNA expression but was downregulated during GnRH differentiation (Kramer and Wray, 2000a). Studies using the mouse GnRH promoter from mice have identified several transcriptional promoter regions that could regulate the expression of GnRH (Iyer et al., 2010). Similar studies have been done in immoraltilized GnRH cell lines (Kim et al., 2011) and in tilapia (Kitahashi et al., 2005). Yet no transcription factor found is restricted to the GnRH cell population. Supporting the need for multiple factors is the work by Lund et al. (2016) on human pluripotent stem cells. In this work, the authors were able to differentiate stem cells to neural progenitor cells expressing FOXG1, EMX2, and PAX6 by dual SMAD inhibition, and then GnRH expressing cells by treating with FGF8, followed by a Notch inhibitor. Thus, a combination of transcription factors and molecular signaling appear to play pivotal roles in the spatiotemporal development of GnRH cells. However, recent work in zebrafish suggests Islet-1 as a potential early marker of GnRH cells (Aguillon et al., 2018). In addition to its expression in the brain and motor neurons of spinal cord (Ericson et al., 1992), Islet-1 was reported in the OP, exclusively in GnRH3 precursor cells (GnRH3 in zebrafish is the ortholog of mouse GnRH1, referred to as GnRH in this paper (reviewed in Forni and Wray, 2012). To determine the origin of these Islet-1/GnRH cells, transgenics and fate mapping experiments were done, and identified solely pre-placodal ectoderm origins and no neural crest derived lineage, consistent with some studies in zebrafish and chicken embryos (reviewed in Forni and Wray, 2012; Steventon et al., 2014; Suzuki and Osumi, 2015). However, the Islet-1 data directly opposes data which suggested that zebrafish GnRH3 derived from SOX10-dependent neural crest (Whitlock, 2005a) and work in mouse that reported up to 30% of GnRH cells are of neural crest origin (Forni et al., 2011). Thus, whether Islet-1 marks the entire GnRH population or only the ectodermally derived cells needs to be further examined in multiple species.

*Fibroblast growth factor* (*FGF*) signaling plays diverse roles during embryonic development, including embryonic patterning and neural development. In the developing OP, FGF8 is expressed in the respiratory epithelium and is required for craniofacial development (Kawauchi et al., 2005; Forni et al., 2013). In FGF8 mutants, OE structures failed to form (Kawauchi et al., 2005). FGF activity prevents prospective placodal cells from acquiring epidermal fate but it alone is not sufficient to specify sensory epithelium (Sjodal et al., 2007; Maier et al., 2010). Loss of FGF8 also affects GnRH development. FGF8 hypomorphic mice (with 50% retention of FGF8 expression), which only survive up to birth, lack GnRH neurons and fibers in the brain (Falardeau et al., 2008). Work in chicken indicates that FGF8 maintains the undifferentiated nature of progenitor cells (Sabado et al., 2012; Chung et al., 2016). This suggests that the timing of FGF8 signaling is important for GnRH neuron formation, and that expression in the OP region must be downregulated before differentiation of GnRH neurons occurs. As one would predict, FGFR1, the receptor for FGF8, is important in OE development. Acute pharmacological inhibition of FGFR1 between E9.5 and E10.5 in mouse resulted in smaller OE, less invagination, and a reduction in expression of critical OE transcriptional regulators PAX6 and NGN1. Additionally, there was reduction in cell proliferation, increase in cell death and reduction in cranial nerve axon growth (Karpinski et al., 2016). Studies in chick embryo explants revealed that FGFR1 uses PI3K pathway, particularly the p110-alpha isoform to regulate GnRH migration and olfactory sensory neuronal outgrowth (Hu et al., 2013). Loss of FGFR1 in mice results in disruption of OB development and a reduction in GnRH neuron numbers (Chung and Tsai, 2010). Mutations in components of the FGF signaling pathway (FGF8, FGF17 and FGFR1) have been identified in KS patients (Section “Spectrum of Genetic Disorders in GnRH Cell Deficiency and Kallmann Syndrome”).

Bone Morphogenetic Protein (BMP), a member of the transforming growth factor (TGF)-β superfamily, plays an important role in formation of the cranial placodes and neurogenesis in the OE. BMP signaling promotes formation of epidermis at the expense of neural induction and promotes respiratory epithelial cell fates (Maier et al., 2010). BMP is regulated by Noggin, a signaling protein involved in patterning and formation of dorsal structures. Our group has shown that BMP originates from nasal mesenchyme, and its expression, along with antagonist Noggin, is crucial in defining neuronal versus epidermal fates in the developing OP (Forni et al., 2013). Moreover, the combination of BMP inhibition with FGF signaling is required for inducing olfactory cell fates (Sjodal et al., 2007; Maier et al., 2010; Forni and Wray, 2015). GnRH neurons form in the VNO at the border between non-neural and neural epithelium, adjacent to mesenchymally expressed Noggin (Forni and Wray, 2015). Changes in BMP and Noggin (via loss of FGF8) also alter the neurogenic pattern of the OE and caused a reduction in the number of GnRH neurons in the developing embryo (Forni et al., 2013).

Wingless-Int (Wnt) signaling, patterns the anterior–posterior axis of the developing embryo and must be antagonized (along with BMP antagonism and FGF activation) for neural induction, cranial placode formation, and cell specification. Wnt is expressed in neural crest derived cells, including OECs and some progenitors, neurons and sustentacular cells of the OE (Forni et al., 2011; Katoh et al., 2011; Wang et al., 2011; Forni and Wray, 2012). In the canonical Wnt (beta-catenin dependent) pathway, Wnt binds to its transmembrane receptor Frizzled (Frz), which stabilizes beta-catenin and directs gene expression. Canonical Wnt is involved in OSN axon growth through various Wnt and Frz family members (Rodriguez-Gil and Greer, 2008). Activation of canonical Wnt signaling is also necessary and sufficient to drive the transition of horizontal basal olfactory cells from a resting to an activated neurogenic state to generate more OSNs. Additionally, loss of Wnt via beta-catenin knockout mice led to premature differentiation of progenitors and ultimately OSNs, while gain of function maintained a progenitor state (Fletcher et al., 2017).

Retinoic Acid (RA) signaling is crucial for craniofacial development. RA inhibits differentiation and maintains self-renewing progenitors before they commit to neurogenesis (Sabado et al., 2012). RA signaling is often analyzed via retinaldehyde dehydrogenases (RALDHs), which convert the retinaldehyde intermediate (retinal) to active RA. RALDH3 is the only member expressed within the neuroepithelium of the developing OE. RALDH3 mediates RA activity in the invaginating olfactory pit (Kawauchi et al., 2004). RA signaling also coordinates with FGF during OE development. RA and FGF pathways oppose each other to pattern the OP and to regulate the transition from self-renewing to neurogenic progenitor cells (Tucker et al., 2010). Inhibition of FGF signaling expands RALDH3 (Kawauchi et al., 2005). In chick, RA represses the formation of GnRH precursors at the time of specification but does not interfere with their subsequent development (Sabado et al., 2012).

## Migration of GnRh Neurons From the Nose to the Brain

**FIGURE 3:**
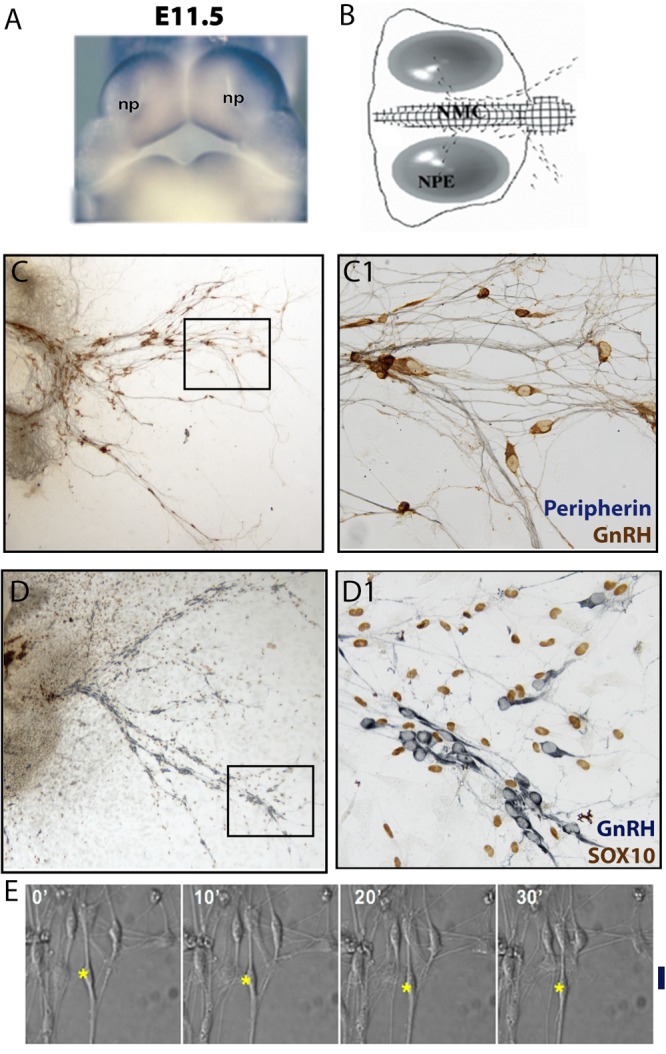
Nasal explant model and migration assay. **(A)** Picture of E11.5 mouse embryo showing the location of the nasal pits (NP). **(B)** Schematic of a nasal explant after 4 days *in vitro* (div); nasal midline cartilage (NMC) and olfactory/VNO epithelium (NPE). GnRH neurons (black dots) migrate out of the nasal pit, toward the NMC, and then into the explant periphery. **(C)** 4div explant stained for peripherin (blue, marking olfactory axons) and GnRH (brown) **(D)** 4div explant stained for GnRH (blue) and SOX10 (brown, marking olfactory ensheathing cells nuclei). Boxed areas are magnified in panels **(C1,D1)**, respectively. **(E)** Example of migration assay using a 4div nasal explant. GnRH cell movement is recorded within a 30 min time frame. ^∗^, nucleus location of GnRH neuron; Black line, linear distance moved after 30 min.

Migration of GnRH cells in nasal regions is intimately entwined with both olfactory axon outgrowth and OEC migration. Many molecules directing this process have been identified and will be discussed below. Much less is known about the migration of GnRH cells once they cross into the forebrain and turn caudally toward the hypothalamus, as well as mechanisms that stop their migration and allow them, from multiple rostrocaudal locations, to then target their axons to the median eminence. Several model systems exist to study migration of the GnRH cells. These include *in vivo* analysis using different species, *in situ* slice preparations, immortalized cell lines, and explant models. The latter, described below, has been used by several groups and takes advantage of the embryonic origin of the GnRH cells, allowing one to study neuronal movement in primary GnRH cells devoid of brain tissue while maintaining many of the components surrounding the GnRH cells *in vivo* (Figure 3). *In vivo*, slice and explants models are feasible, because of the early expression of GnRH, allowing one to monitor their development.

**FIGURE 4:**
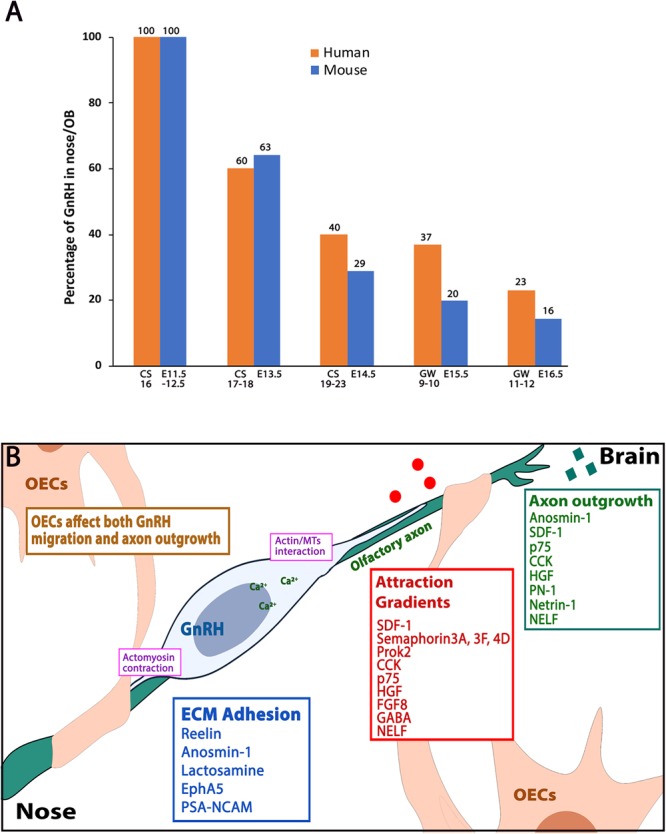
Human-mouse GnRH distribution plot and molecules affecting GnRH neuronal migration. **(A)** Plot showing percentage of GnRH neurons that reside in nose/OB area between human (orange bars) and mouse (blue bars) at five developmental windows. CS: carnegie stage, GW: gestation week, E: embryonic stage. Data adapted from Casoni et al. (2016) and Wray et al. (1989b). **(B)** Schematic showing a GnRH neuron (blue) along the olfactory axon bundles (green), which are ensheathed by OECs (beige). Categories of axon outgrowth cues (green box), migration attraction gradients cues (red box) and ECM adhesion molecules (blue box) are indicated. Actin/microtubule interactions and actomyosin contractions (pink boxes) occur at the leading process and the trailing process of the migrating GnRH neuron.

GnRH neuronal migration was first categorized as axophilic/neurophilic since they used olfactory sensory axons/terminal nerve fibers to reach the forebrain (Wray et al., 1989a; Taroc et al., 2017). However, it is now clear that the GnRH cells in nasal regions migrate on tracks composed of (1) a subset of olfactory axon bundles originating from sensory cells in the VNO, (2) terminal nerve bundles/transient axons, (3) blood vessels, (4) other neurons and (5) OECs (Wray, 2002b, 2010; Forni and Wray, 2012). Although many diverse components are involved in the migration of GnRH cells, the overall process is maintained across vertebrates, as recent studies highlight the similarities between development of this system in mouse and human (Casoni et al., 2016). It should be noted however, that the location of the cells can vary depending on the species and may be a consequence of the species’ specific brain development as the cells migrate in. Yet, if one compares the development of GnRH cells in mouse and human, one finds that the GnRH cells become postmitotic early, rapidly increase in number and begin their migration quickly (Figure 4A; Wray et al., 1989b; Casoni et al., 2016). No sex differences are seen and although most GnRH cells integrate into the hypothalamic-pituitary axis and reside within the preoptic area (POA)/hypothlamus, scattered cells are seen in other regions including the hippocampus, cerebral cortex, and amygdala (Hoffman et al., 1992; Casoni et al., 2016). Although species differences in GnRH cell location exist, there are two unifying principles of GnRH cell distribution. First, they avoid defined cytoarchitectonically identified nuclei and second, they lie in a continuum extending from the olfactory bulbs, at varying distances, through the midline septum to the ventral hypothalmus. In mice, primates and humans, a relatively large population of GnRH cells in the ventral telencephalon has been identified (Quanbeck et al., 1997; Casoni et al., 2016), however, in mice and primates, these cells differ from the neuroendocrine GnRH expressing cells in their reactivity to anti-mammalian GnRH antisera (Quanbeck et al., 1997; Casoni et al., 2016; Wray personal observation). In primates, this population was shown to originate from the olfactory placode before the formation of the olfactory pit, and migrate into the brain along the olfactory nerve, rather than the terminal nerve, and eventually settle in striatal and limbic structures. In both mice and primates, these cells are smaller than neuroendocrine GnRH cells. In mice, these cells are not detected pre- or post-natally using *in situ* hybridization for GnRH mRNA (Wray personal observation) and their fate remains unknown. All data presented below refers to the neuroendocrine GnRH cell population.

### Formation of Migratory Tracks

In mammals, GnRH neurons migrate along axons of cells that reside within the VNO (Figure 1). These axons, together with OECs and non-GnRH neurons, form a track/bundle – this is the migratory pathway on which GnRH cells move from the VNO to the cribriform plate. This bundle converges with axons and OECs from the mOE and targets the cribriform plate. Once through the cribriform plate and meninges, OECs and axons from sensory neurons in mOE and VNO turn rostrally to enter the olfactory bulb. GnRH cells on the other hand, follow a transient pathway/nervus terminalus (TN), caudally turning away from the olfactory bulb toward the hypothalamus (Wray et al., 1994; Tobet and Schwarting, 2006; Taroc et al., 2017). This nerve is transient in some species including humans (Fuller and Burger, 1990; Muller and O’Rahilly, 2004; Whitlock, 2004). VNO derived fibers in the nasal region are positive for TAG-1 (transiently expressed axonal surface glycoprotein, (Yoshida et al., 1995) and DCC (Deleted in Colorectal Carcinoma, netrin-1 receptor) (Deiner et al., 1997). The expression of these two markers is not detectable on the caudal trajectory. However, knocking out DCC results in the caudal trajectory turning toward the cortex (Schwarting et al., 2001) and misdirected GnRH cells (Deiner and Sretavan, 1999). In the olfactory sensory system of some vertebrates (zebrafish, xenopus, etc.) (Demski and Schwanzel-Fukuda, 1987; Muske and Moore, 1988; Mousley et al., 2006; Zhao et al., 2013), the TN remains after birth. Taroc et al. (2017) suggested that mouse GnRH neurons can properly migrate via TN fibers without the development of the OB and olfactory/VNO axonal connections. Further investigations are needed to distinguish what extracellular cues are guiding the TN outgrowth specifically. Some of the molecules guiding olfactory axons outgrowth are listed in Table 1.

**Table 1:** Guidance molecules involved in embryonic olfactory axon outgrowth.

Molecule	Function on Olfactory Axons	References
Nasal embryonic LHRH factor (NELF)	NELF was expressed in OSN and GnRH during development.	Kramer and Wray, 2000b
Fibroblast growth factor receptor 1 (FGFR1)	FGFR1-associated PI3K pathway was required for OSNs projection to the olfactory bulbs.	Hu et al., 2013
SDF-1/CXCR4	Olfactory axon outgrowth was impacted in CXCR4 KO animals.	Casoni et al., 2012
P75	An absence of GAD67+ olfactory fibers in p75KO mice. Chronic Inhibition of p75 caused a reduced fasciculation of olfactory axon.	Raucci et al., 2013
Cholecystokinin (CCK)	CCK reduced olfactory axon outgrowth when applied exogenously.	Giacobini et al., 2004
Hepatocyte growth factor (HGF)	Axon outgrowth was reduced when HGF-neutralizing antibody was applied to explants.	Giacobini et al., 2007
Protease nexin-1 (PN-1)	In chicken embryo, PN-1 accelerated biochemical differentiation of olfactory axons.	Drapkin et al., 2002
Netrin-1	Misexpression of Netrin-1 caused subset of OAs to extend, but no GnRH were observed on these fibers.	Murakami et al., 2010

Olfactory ensheathing cells migrate along the olfactory axons through the nasal region, ensheathing the olfactory axon bundles going into the OB, and also reside in the external layer of the OB (Doucette, 1990; Cummings and Brunjes, 1997; Barnett, 2004; Miller et al., 2010). They are not detected on the caudal projection that GnRH neurons follow toward the hypothalamus. However, in nasal regions they are in close proximity to GnRH cells (Geller et al., 2013; Raucci et al., 2013; Dairaghi et al., 2018). OECs express p75NTR (nerve growth factor receptors) (Yan et al., 2001). Signaling via p75NTR has been shown to regulate OEC migration (Raucci et al., 2013). p75NTR KO mice showed reduced olfactory axons and OECs in the nasal region (Raucci et al., 2013). A subpopulation of GnRH cells also express p75NTR. Although ablation of p75NTR did not alter the total number of GnRH neurons, a small population of GnRH neurons migrated further in the hypothalamus in the p75NTR KO mice (Raucci et al., 2013). Whether this change is direct, or indirect via changes in OECs is unclear. *Sox10* is expressed by migrating neural crest cells and required for the specification and differentiation of neural crest-derived Schwann cells and normal OEC differentiation (Barraud et al., 2013). Disruption of OECs by *SOX10* mutations caused embryonic olfactory axons to accumulate in the ventromedial nerve layer in the OB, and slower migration of GnRH neurons into the forebrain was reported at early ages (Barraud et al., 2013). *SOX10* mutations have been identified in KS patients (see below).

OSNs express distinct receptors and project to specific glomeruli in the main olfactory bulb or accessory olfactory bulb (Sakano, 2010). Independent of the location of the OSN, or their target, all sensory axons must traverse the cribriform plate to gain access to the central nervous system (CNS). This process is also critical for GnRH cells to enter the brain. Thus, outgrowth of fibers from the mOE can impact GnRH cell migration specifically at the cribriform plate. This was observed in Fez family zinc-finger protein 1 (*Fezf1*) KO mice. FEZF1 is a transcription factor that is essential for olfactory development and olfactory axon projection to the olfactory bulb (Watanabe et al., 2009). Experiments indicate that FEZF1 is required for the penetration of olfactory axons through the CNS basal lamina and thus for them to innervate the OB. When this does not occur, an axonal tangle results which halts GnRH neuronal migration as well. A homozygous loss of function in FEZF1 has been identified in KS patients, who showed attenuated OBs and absence of puberty (Kotan et al., 2014) (see Section “Spectrum of Genetic Disorders in GnRH Cell Deficiency and Kallmann Syndrome”).

### GnRH Cell Migration

While GnRH neurons have a unique migration route (from outside the brain), like other types of migrating neurons they require cell–cell adhesive mechanisms to remain on the olfactory axon bundles, as well as a variety of guidance molecule gradients (secreted peptides, growth factors, cytokines and chemokines etc.) to direct them. Nasal explant systems have been developed to study GnRH migration (Terasawa et al., 1993; Fueshko and Wray, 1994; Kanaho et al., 2009). These explant models: (1) contain a large number of primary GnRH neurons, olfactory sensory neurons and OECs; (2) allow one to perform single cell tracing and imaging; (3) are accessible to both molecular and pharmacological manipulations (Figure 3) (Wray et al., 1994; Klenke and Taylor-Burds, 2012). Although no brain is present, the GnRH cells mature in this model with respect to both processing of the peptide, extension of processes and cellular properties (Wray et al., 1994; Klenke and Taylor-Burds, 2012; Constantin, 2017), suggesting that the cells in explants are equivalent to their counterparts *in vivo*, aging appropriate. In explants generated from mice, olfactory axon outgrowth, OEC migration and GnRH cell migration occur between days 1 and 7 (Wray et al., 1996). However, after day 4, migration rates decrease (Casoni et al., 2016), similar to what would occur *in vivo*, as many of the cells have reached their final location by E15.5-E18.5. Certainly, when possible, parallel *in vivo* studies are run. Often the results are comparable, but not always, leaving one to search for redundancy *in vivo* that might not be present in explants or a maturation event that was not triggered in the explant. However, in the mouse nasal explant model, GnRH neurons migrate at 12–16 μm/h, and exhibit saltatory movement (Casoni et al., 2012), similar to GnRH neurons in studied in slices (Bless et al., 2005), and glial fiber mediated migration *in vivo* (Edmondson and Hatten, 1987). Thus, studying GnRH migration in primary nasal explant using techniques like live cell imaging, *post hoc* immunolabeling, calcium recording, and pharmacological/genetic manipulation has enhanced our understanding of how GnRH neurons develop and mature.

For a cell to migrate properly, the coordination of extracellular guidance cues and intracellular cytoskeletal elements is required. Actin filaments, microtubule bundles and centrosomes, plus cytoskeletal associated proteins are involved in migration (Coles and Bradke, 2015). In a migrating cell, cortical actin filaments are located underneath the plasma membrane and interact with microtubules, via multiple actin/tubulin binding proteins (Rodriguez et al., 2003). Intracellular signals control the extension and retraction of microtubule bundles, which creates a “pulling force.” Saltatory movement has been characterized in many types of migrating neurons (Schaar and McConnell, 2005), including GnRH neurons (Casoni et al., 2012). The sequence of events in migrating GnRH cells includes: (1) the leading process extends and contacts on the scaffolding that supports the migration (glial fiber, sensory axons, ECM, etc.), (2) the centrosome moves forward into a bulging area in the leading process, (3) the microtubule bundles form a cage around the nucleus, and the “pulling force” from actin/microtubule interaction pulls the nucleus toward the centrosome, and (4) myosin II, at the rear end of the moving cell, generates a “pushing force” by actinomyosin contraction (Hutchins et al., 2013). The saltatory movement pattern of GnRH neurons is similar to radial migrating cells in the developing cortices (Edmondson and Hatten, 1987; Schaar and McConnell, 2005) and tangential migrating neurons in medial ganglionic eminence (Bellion et al., 2005). Ca^2+^ signaling is known to be involved in the transduction of extracellular cues and conformational change of actin mesh (Komuro and Kumada, 2005; Fahrion et al., 2012). Hutchins and Wray (2014) showed that by blocking or enhancing Ca^2+^ signaling, GnRH neuronal migration was inhibited or increased accordingly. Furthermore, live actin tracing in migrating GnRH neurons revealed that leading process actin mesh translocation and rear actin contraction corresponded with Ca^2+^ signaling (as observed in glial mediated neuronal migration, (Solecki et al., 2009), and microtubule plus end capturing in the actin mesh was reduced when Ca^2+^ signaling was inhibited via the calcium channel (IP3 receptors) blocker, 2-APB (Hutchins and Wray, 2014). Cortactin is known to promote F-actin polymerization and cellular motility in fibroblast (Nakane et al., 2012). Sirtuin1 (a NAD-Dependent Protein Deacetylase Sirtuin-1) deacetylates cortactin and was in GN11 cells (an immortalized GnRH cell line). Sirtuin1 deficient mice have an IHH phenotype due to defective GnRH neuronal migration (Di Sante et al., 2015). Sirtuin1-Cortactin co-localization in the GN11 cells was FGF8/FGFR1 dependent, suggesting that FGF signaling maybe involved in GnRH neuronal migration as well as OP development.

### Factors Influencing GnRH Cell Migration (Figure 4B)

The semaphorin (SEMA) proteins, acting as guidance cues, are necessary for appropriate development of the GnRH/olfactory system. Disruption of three class 3 SEMAs, SEMA3A, SEMA3F and SEMA3E perturbs GnRH development (Messina and Giacobini, 2013; Cariboni et al., 2018). SEMA3A and SEMA3F, are involved in proper GnRH migration. SEMA3A binds to neuropilin (NRP) 1 and 2 (Kolodkin et al., 1997; Reza et al., 1999). In mice, lack of SEMA3A altered VNO nerves and inhibited GnRH cell migration (Cariboni et al., 2011) while, lack of either SEMA3E or its receptor, PLXND1, increased apoptosis of GnRH neurons and reduced GnRH terminals in the median eminence (Cariboni et al., 2015). SEMA4D is expressed on the GnRH cell migratory pathway, with higher expression in the hypothalamic area (Giacobini et al., 2008). SEMA4D binds to PLEXINB1, which is robustly expressed in the OP during development (Perala et al., 2005) as well as on migrating GnRH cells (Giacobini et al., 2008). *PlexinB1* KO mice show a delay in GnRH migration and reduced GnRH cells in adult brains. *SEMA3A, SEMA3E* and *SEMA7A* mutations have been reported in KS patients (Hanchate et al., 2012; Young et al., 2012; Kansakoski et al., 2014) (see below).

Reelin is an ECM glycoprotein. Reelin has a canonical pathway where it binds to low-density lipoprotein receptor (VLDLR) and apolipoprotein R receptor 2 [APOER2, (Hiesberger et al., 1999)], and eventually leads to the degradation of Disabled 1 (DAB1). Non-canonical pathways of reelin (independent of DAB1 mechanism) were reported in migrating interneurons in the olfactory bulb (Hellwig et al., 2012), lymphatic vascular development (Lutter et al., 2012) and hypothalamic GnRH neurons (Cariboni et al., 2005). However, a recent study (Dairaghi et al., 2018) showed that both VLDLR, APOER2 and DAB1 were, in fact, expressed in GnRH cells as well as in OECs. Although immunodepletion of Reelin using an explant model inhibited GnRH and OEC migration, no change in GnRH or OEC distribution was detected in homozygous reelin mutant mice.

Several G protein-coupled receptors and their ligands modulate GnRH cell movement. Stromal cell derived factor-1 (SDF-1) and its receptor chemokine receptor type 4 (CXCR4) regulate neuronal migration and axonal pathfinding in a number of brain regions (Stumm and Hollt, 2007). CXCR4 is detected on cells in the OE and on GnRH neurons during development. An SDF-1 signal is seen in GnRH cells as well as cells in the midline cartilage and the mesenchyme surrounding the OE (Schwarting et al., 2007; Toba et al., 2008). Both CXCR4 and SDF-1 are also expressed on the olfactory nerves. *Cxcr4* homozygous KO animals die at E18.5, but younger embryos have disrupted GnRH cell migration and inhibited olfactory axon outgrowth (Schwarting et al., 2007; Toba et al., 2008). SDF-1/CXCR4 signaling accelerates the movement of GnRH neurons in explants, utilizing GIRK channels to promote GnRH movement (Casoni et al., 2012). Prokineticin 2 (PROK2) has been reported to be associated with olfactory bulb development and GnRH cell migration (Ng et al., 2005). KO mice of PROK2 or its receptor PROKR2 show a decrease in GnRH cells in the forebrain (Matsumoto et al., 2006; Zhao et al., 2019). However, *ProkR2* KO mice have a reduced OB size, whereas *Prok2* KO mice have normal sized OB (Martin et al., 2011). Mutations of *PROK2* and *PROKR2* are seen in patients with KS (Dode et al., 2006). Expression of the neuropeptide cholecystokinin (CCK) and its receptors CCK-1R and CCK-2R are seen in developing OE, olfactory axons, and the VNO (Giacobini et al., 2004). GnRH neurons express CCK-1R but not CCK-2R. Exogenous application of CCK to nasal explants reduced GnRH migration and olfactory axon outgrowth. In *Cck1R* KO mice, the total number of GnRH cells was identical in wild-type and mutant mice at E14.5. However, the number of GnRH neurons within the forebrain was significantly greater in *Cck1R*^-/-^ embryos, consistent with an accelerated migratory process. These results indicate that CCK provides an inhibitory influence on GnRH neuronal migration, contributing to the appropriate entrance of these neuroendocrine cells into the brain. G protein-coupled receptor 37 (GPR37 or PAEL-r) and its ligand prosaposin (PSAP) have recently been shown to be expressed in developing GnRH neurons and OECs. Inhibition of GPR37 attenuates migrations of GnRH neurons and OECs, and acute migration assay shows that application of a GPR37 agonist accelerates GnRH migration. In GPR37 KO animals, GnRH development and migration were delayed. GPR37 KO animals also showed an altered OB development, with a thinner olfactory nerve layer present in adults (Saadi et al., 2019).

Hepatocyte growth factor (HGF), Gamma-amino butyric acid (GABA) and nasal embryonic LHRH factor (NELF) have also been shown to alter GnRH cell movement. HGF is a cytokine that binds to its receptor MET tyrosine kinase (Ebens et al., 1996; Giacobini et al., 2007). HGF is expressed in nasal mesenchyme during embryonic development with an increased concentration toward the nasal forebrain junction (Sonnenberg et al., 1993; Giacobini et al., 2007). MET is expressed in migratory GnRH neurons, but not in post-migratory GnRH cells (Giacobini et al., 2002). In mice, KO of tissue-type plasminogen activator (tPA, an HGF activator) reduced the total number of GnRH cells (Giacobini et al., 2007). In nasal explants, blocking HGF with a neutralizing antibody caused a reduction in GnRH cell migration and olfactory axon outgrowth. This effect was then rescued by exogenous application of HGF. During development GABA acts as an excitatory neurotransmitter. During GnRH cell migration, a subset of GnRH neurons (30%) express GABA (Tobet et al., 1996), as do the olfactory axons and migratory cells along the migratory route (Wray et al., 1996). Expression of GABAAR is seen on the majority of GnRH neurons, but with heterogeneous subunit compositions (Fueshko et al., 1998; Bless et al., 2000). The GABAAR agonist Muscimol inhibited GnRH cell migration and reduced GnRH fiber extension. The GABAA antagonist Bicucullin disorganized GnRH cells in the forebrain and dissociated GnRH cells from olfactory fibers (Bless et al., 2000). Activation of GABAAR in explant resulted in increased Ca^2+^ signaling and membrane depolarization in GnRH neurons and promoted GnRH migration (Moore and Wray, 2000), with GABA signaling tending to orient cell movement (Casoni et al., 2012). Kramer and Wray first identified nasal embryonic LHRH factor (NELF, now termed NSMF) in 2000, where NSMF expression was detected in mouse nasal epithelia, migrating GnRH neurons and olfactory axons (Kramer and Wray, 2000b). Both olfactory axon outgrowth and GnRH migration were attenuated after applying antisense oligonucleotides. Studies in *Nsmf* KO mouse are conflicting. One study reported that in females there was a decrease in GnRH number, as well as an impaired fertility (delayed vaginal opening, decreased uterine size, and reduced mean litter size). No delayed puberty was observed in males (Quaynor et al., 2015). However, in a second *Nsmf* KO model, no changes in GnRH neuronal number or reproductive function was observed (Spilker et al., 2016). To date, six variant transcripts of *Nsmf* have been identified (Quaynor et al., 2014). Further studies are needed to identify whether splice variant expression might account for these differences. A *NSMF* heterozygous missense (T480A) (Miura et al., 2004) and three cases of *NSMF* digenic mutations (Pitteloud et al., 2007; Xu et al., 2011) have been reported associated with KS patients.

Migrating neurons following a pathway must attach and subsequently detach to facilitate saltatory movement. A variety of adhesive molecules are implicated in this mechanism for GnRH neurons. NCAMs are surface associated adhesion molecules. PSA-NCAM is expressed in developing nervous system regions that are associated with migrating neurons and synaptogenesis (Kiss and Rougon, 1997). PSA-NCAM is seen on GnRH neurons and along the migratory pathway and is suggested to be involved in the pathway formation, as well as guiding GnRH neurons from the VNO to the brain (Schwanzel-Fukuda et al., 1992, 1996; Yoshida et al., 1995). PSA-NCAM is also seen on OECs (Franceschini and Barnett, 1996), though no functional studies have been done to date. The function of PSA-NCAM is believed to be highly dependent on the sialic acid polymer chain (Yang et al., 1994). Experiments in chick embryos examining a late migratory stage showed that removal of PSA (via Endo N, a PSA specific enzyme) from the PSA-NCAM molecule resulted in a significant reduction of caudal turning GnRH neurons in the forebrain, however, the morphology of the caudal turning fibers did not change (Murakami et al., 2000). Notably, PSA-NCAM expression is not detectable when GnRH neurons reach their destination (Murakami et al., 1991).

Glycoconjugates containing terminal lactosamine at the carbohydrate chains have been seen in neurons within the OE and the VNO. Beta1,3-N-acetylglucosaminyltransferase-1 (B3GNT1), an enzyme that regulates the synthesis of lactosamine, is expressed in cells in the OE and VNO, and migratory GnRH neurons. Lactosamine expression is high on migrating GnRH neurons but decreases dramatically in post-migratory GnRH cells. In *B3gnt1* KO mice, more GnRH neurons were seen at E15 in olfactory region while fewer GnRH neurons were detected in the forebrain (Bless et al., 2006). Ephrins are a family of cell surface proteins that binds to Ephrin receptor tyrosine kinases. In a mouse model with overexpressed subtype 5 Ephrin type-A receptor (EPHA5), GnRH neuronal migration was disrupted. It is proposed that the overexpression of EPHA5 caused abnormal adhesion of GnRH/olfactory axons (Gamble et al., 2005).

## Spectrum of Genetic Disorders in GnRh Cell Deficiency and Kallmann Syndrome

Idiopathic hypogonadotropic hypogonadism (IHH) is a rare disorder that results from the failure of the normal episodic GnRH secretion, leading to delayed puberty and infertility. Since GnRH neurons arise in the olfactory placode and share developmental events with the olfactory system, approximately 60% of IHHs are accompanied by an impaired sense of smell (anosmia), which is categorized as KS. The remaining 40% of IHHs patients show a normal sense of smell (nIHH) (Sedlmeyer and Palmert, 2002; Lewkowitz-Shpuntoff et al., 2012). Notably, some mutations associated with IHH can cause either KS or nIHH, suggesting other mutations or underlying dysfunctions may be present. About 60% of patients with IHH are sporadic cases without family history, while ∼40% are familial cases who already have family member(s) with associated symptoms (Seminara et al., 1998; Oliveira et al., 2001). With the development of medical treatment for IHH, patient fertility rates have increased, increasing offspring and the proportion of familial cases among the IHH patient population. This phenomenon emphases the importance of understanding the underlying genetics of IHH to develop genetic diagnosis and genetic counseling for patients.

### Clinical Features of IHH

#### Reproductive Phenotype

Idiopathic hypogonadotropic hypogonadism can be diagnosed at different developmental stages. Some patients with IHH show clinical features such as cryptorchidism and/or microphallus at birth, and may have biochemical evidence of low gonadotropins and sex steroids during the minipuberty (occurs at 3–6 months in humans, and is accompanied by a transient activation of the HPG axis and higher levels of gonadotropins/sex steroids) (Tapanainen et al., 1981; Kuiri-Hanninen et al., 2011a,b). However, most patients with IHH do not present with neonatal features and can only be diagnosed at the time of puberty. In either sex, the patients cannot develop secondary sex characteristics. In females this includes growth of body hair, start of menstruation (primary amenorrhea), and enlargement of breasts. In males this includes growth of facial and body hair, deepening of voice, enlargement of the scrotum and testes followed by growth of the penis (Aksglaede et al., 2009; Herman-Giddens et al., 2012; Boehm et al., 2015).

#### Olfactory Phenotype

Variable phenotypes and penetrance of olfactory symptoms are observed in IHH patients. KS patients can exhibit an absence of smell (anosmia) to decreased sense of smell (hyposmia) (Pitteloud et al., 2006; Trarbach et al., 2006; Bianco and Kaiser, 2009). Olfactory function is evaluated by history and by formal diagnostic smell tests (Doty, 2007) which allow classification of the patient as anosmic, hyposmic, or normosmic. In families with KS affected members, anosmia has been reported to be present as an isolated symptom (Dode et al., 2003; Pitteloud et al., 2006). In addition to attenuated olfactory function, anatomical malformations of the olfactory bulbs are frequently observed in MRIs of KS patients, with partially developed (hypoplasia) or complete absence (aplasia) of the olfactory bulb reported (Truwit et al., 1993; Sato et al., 2004).

#### Other Phenotypes

Kallmann syndrome patients can exhibit a wide spectrum of non-reproductive features resulting from aberrant developmental events including midline anomalies (a cleft lip/palate, high-arched palate, and dental agenesis) (Dode et al., 2003; Falardeau et al., 2008; Kim et al., 2008; Jongmans et al., 2009), ear impairment (hearing loss, external ear anomalies, and semicircular canal dysplasia) (Dode et al., 2003; Zenaty et al., 2006; Jongmans et al., 2009), eye problems (coloboma of the eye and abnormal eye movements) (Kim et al., 2008; Jongmans et al., 2009), unilateral renal agenesis (Wegenke et al., 1975; Georgopoulos et al., 2007), abnormalities of bones in limb and digit, and bimanual synkinesis (involuntary movement of one hand that are mirrored by the other hand) (Quinton et al., 2001; Costa-Barbosa et al., 2013).

### Molecular Genetics of KS

The appearance of high throughput sequencing techniques and advances in neuroimaging technology have led to an impressive advance in the molecular genetics of KS in the past 30 years, from the first finding of a causative gene associated with KS, *KAL1* (*ANOS1*), in 1991 (Franco et al., 1991; Legouis et al., 1991; Bick et al., 1992; Hardelin et al., 1992) to 25 genes today (see Table 2). It was believed that a mutation in a single gene was sufficient to cause KS (monogenic disorder) and the penetrance of the mutation in a patient resulted in phenotypic variability within and across families. However, recent studies show the existence and prevalence of oligogenism in KS patients, indicating that a combination of mutations or rare variants on two or more genes can underlie the disease (di/oligogenic disorder) (Sykiotis et al., 2010; Miraoui et al., 2013). In fact, oligogenism is now thought to explain the complex variability in some of the clinical phenotypes observed in IHH patients in general (Dode et al., 2006; Martin et al., 2011; Cox et al., 2018). The known genes involved in KS can be categorized by their inheritance pattern (X-linked, autosomal dominant, autosomal recessive, oligonism, and *de novo*) as shown in Table 2.

**Table 2:** The known causative genes related with idiopathic hypogonadotropic hypogonadism.

Gene	Description	Location	Inheritance pattern	Expression levels (FPKM)			Type of IHH	Functions involved in IHH
				OSN	OEC	GnRH		
*ANOS1*	Anosmin 1	Xp22.31	X-linked	ND^∗^	0.670	ND^∗^	KS	OA outgrowth and GnRH migration
*FGFR1*	Fibroblast growth factor receptor 1	8p11.23	AD/AR/Oligo/*de novo*	7.048	22.639	3.786	KS and nIHH	OE and OB development
*FGF8*	Fibroblast growth factor 8	10q24.32	AD/Oligo	ND	0.050	0.213	KS and nIHH	OE and GnRH development
*FGF17*	Fibroblast growth factor 17	8p21.3	AD/Oligo/*de novo*	0.204	0.004	3.120	KS and nIHH	OE and OB development
*IL17RD*	Interleukin 17 receptor D	3p14.3	AD/AR/Oligo	11.459	0.096	0.022	KS	GnRH development
*DUSP6*	Dual specificity phosphate 6	12q21.33	AD/Oligo	9.080	16.846	8.960	KS and nIHH	NS
*SPRY4*	Sprouty RTK signaling antagonist 4	5q31.3	AD/Oligo	1.218	9.987	2.916	KS and nIHH	NS
*FLRT3*	Fibronectin leucine rich transmembrane protein 3	20p12.1	AD/Oligo	2.591	ND	5.049	KS	OA outgrowth and GnRH migration
*KLB*	Klotho beta	4p14	AD/Oligo/*de novo*	ND	0.074	0.061	KS and nIHH	NS
*SEMA3A*	Semaphorin 3A	7q21.11	AD/Oligo	3.732	0.968	0.465	KS	GnRH migration
*SEMA3E*	Semaphorin 3E	7q21.11	AD	9.442	0.110	0.325	KS	GnRH maintenance
*SEMA7A*	Semaphorin 7A	15q24.1	Oligo	104.814	32.294	1.204	KS and nIHH	GnRH development and migration
*PLXNA1*	Plexin A1	3q21.3	AD/Oligo	90.990	10.843	5.429	KS and nIHH	OB development and GnRH migration
*PROK2*	Prokineticin 2	3p13	AD/Oligo	ND	0.007	4.475	KS and nIHH	OB development and GnRH migration
*PROKR2*	Prokineticin receptor 2	20p12.3	AD/AR/Oligo	0.122	ND	0.329	KS and nIHH	OB development and sexual maturation
*SOX10*	SRY-box 10	22q13.1	AD	3.209	0.003	12.002	KS	OEC and OB development, and GnRH migration
*FEZF1*	Fez family zinc finger protein 1	7q31.32	AR/Oligo	63.541	0.002	2.374	KS	OA projection
*HESX1*	HESX homeobox 1	3p14.3	AD	0.818	0.300	0.023	KS	Olfactory and pituitary development
*TUBB3*	Tubulin beta 3 class III	16q24.3	AD*/de novo*	160.630	126.927	277.850	KS	NS
*NSMF*	NMDA receptor synaptonuclear signaling and neuronal migration factor	9q34.3	Oligo	117.333	18.119	125.393	KS and nIHH	OA outgrowth and GnRH migration
*HS6ST1*	Heparan sulfate 6-*O*-sulfotransferase 1	2q14.3	AD/Oligo	63.521	20.542	6.793	KS and nIHH	NS
*CHD7*	Chromodomain helicase DNA binding protein 7	8q12.2	AD/Oligo/*de novo*	10.888	0.016	7.257	KS and nIHH	OSN and OB development
*WDR11*	WD repeat domain 11	10q26.12	AD	17.794	11.081	5.893	KS and nIHH	OB, GnRH, and pituitary development
*AXL*	AXL receptor tyrosine kinase	19q13.2	AD	0.723	110.512	0.578	KS and nIHH	NS
*CCDC141*	Coiled-coil domain containing 141	2q31.2	AR/Oligo	1.408	0.001	0.394	KS and nIHH	GnRH migration
*OTUD4*	OTU deubiquitinase 4	4q31.21	AR/Oligo	25.376	9.631	3.146	nIHH	NS
*RNF216*	Ring finger protein 216	7p22.1	AR/Oligo	15.862	11.211	7.207	nIHH	GnRH migration
*POLR3A*	RNA polymerase III subunit A	10q22.3	AR	13.189	6.374	1.475	nIHH	NS
*POLR3B*	RNA polymerase III subunit B	12q23.3	AR	5.310	3.455	1.907	nIHH	NS
*PNPLA6*	Patatin like phospholipase domain containing 6	19p13.2	AR	22.132	32.244	6.890	nIHH	NS
*STUB1*	STIP1 homology and U-box containing protein 1	16p13.3	AR	38.359	32.093	666.014	nIHH	NS
*DMXL2*	Dmx like 2	15q21.2	AR	55.794	5.106	3.882	nIHH	GnRH and Gonadotropin secretion
*GNRH1*	Gonadotropin releasing hormone 1	8p21.2	AR/Oligo	1.986	0.332	37010.3	nIHH	GnRH secretion
*GNRHR*	gonadotropin releasing hormone receptor	4q13.2	AR/Oligo	ND	0.280	0.006	nIHH	GnRH action
*KISS1*	KiSS-1 metastasis suppressor	1q32.1	AR	ND	6.396	47.947	nIHH	GnRH secretion
*KISS1R*	KISS1 receptor	19p13.3	AD/AR/Oligo	2.323	0.070	15.173	nIHH	GnRH secretion
*TAC3*	Tachykinin 3	12q13.3	AR/Oligo	ND	0.053	ND	nIHH	GnRH secretion
*TACR3*	Tachykinin receptor 3	4q24	AR/Oligo	ND	ND	0.933	nIHH	GnRH secretion
*LEP*	Leptin	7q32.1	AR	ND	0.005	ND	nIHH	GnRH secretion
*LEPR*	Leptin receptor	1p31.3	AR	ND	52.343	0.304	nIHH	GnRH secretion
*NR0B1*	Nuclear receptor subfamily 0 group B member 1	Xp21.2	X-linked	ND	0.078	0.040	nIHH	Gonads and adrenal cortex development
*FSHB*	Follicle stimulating hormone subunit beta	11p14.1	AR	ND	ND	ND	nIHH	Gonadotropin secretion
*LHB*	Luteinizing hormone beta polypeptide	19q13.33	AR	ND	0.602	0.907	nIHH	Gonadotropin secretion
*LHX4*	LIM homeobox 4	1q25.2	AR	0.159	0.862	ND	nIHH	Pituitary development
*PROP1*	PROP paired-like homeobox 1	5q35.3	AR	ND	ND	ND	nIHH	Pituitary development and Gonadotropin secretion
*PCSK1*	Proprotein convertase subtilisin/kexin type 1	5q15	AR	1.381	0.997	3.016	nIHH	GnRH secretion
*SOX2*	SRY-box 2	3q26.33	AR	23.614	0.081	29.861	nIHH	Pituitary development

*AD, Autosomal dominant; AR, Autosomal recessive; Oligo, Oligogenic; ND, Not detected; NS, Not specified. ^∗^*ANOS1* gene doesn’t exist in the mouse genome.*

### Genes in KS Patients

The genes mutated in KS patients include: *ANOS1, FGFR1, FGF8, FGF17, IL17RD, DUSP6, SPRY4, FLRT3, KLB, SEMA3A, SEMA3E, SEMA7A, PLXNA1, PROK2, PROKR2, SOX10, FEZF1, HESX1, TUBB3, NSMF, HS6ST1, CHD7, WDR11, AXL*, and *CCDC141*. Genes identified in only nIHH patients have a role in the development of the hypothalamic-pituitary axis (*NR0B1*), neuroendocrine physiology of the normal secretion of GnRH (*GnRH1, KISS1, KISSIR, TAC3, TACR3, LEP*, and *LEPR*) or its action on the pituitary (*GNRHR*) (Stamou and Georgopoulos, 2018), and will not be discussed here. The genes identified in patients with KS are primarily neurodevelopmental genes. Notably, if the GnRH cells fail to enter the forebrain, reproductive function will be lost, and the mutation removed from the population. In some of the mouse models examined, a subpopulation of GnRH cells is able to enter the CNS, and subfertility is observed, indicating that redundant systems are present to ensure GnRH cells enter the forebrain and maturation of the reproductive system occurs. The biological role of several KS associated genes have been discussed in the previous two sections. These include *FGFR1, FGF8, SEMA3A, SEMA3E, SEMA7A, PLXNA1, PROK2, PROKR2, SOX10, CCDC141, FEZF1, ANOS1* and *NSMF*. Below the remaining KS genes are discussed and clinically relevant sections are added for some of the genes mentioned earlier.

#### ANOS1 (KAL1)

*ANOS1* was first identified as one of multiple genes lost in a large deletion on the X chromosome of a patient with that showed multiple clinical phenotypes such as short stature, chondrodysplasia punctata, intellectual disability, ichthyosis and KS (Franco et al., 1991; Legouis et al., 1991; Bick et al., 1992; Hardelin et al., 1992). Anosmin-1 is an ECM glycoprotein and a product of *ANOS1* gene (de Castro et al., 2017). Anosmin-1 contains four consecutive fibronectin type III domains (FNIII), known to be present in the proteins involved in cell adhesion, tyrosine kinases and phosphatases (Franco et al., 1991; Hu and Bouloux, 2011). Unfortunately, the corresponding region of the X chromosome containing *ANOS1* was lost in mouse. However, experiments in Medaka showed, consistent with the X-KS phenotype, antisense knockdown of the Medaka KAL1 ortholog resulted in the disruption of forebrain GnRH neuronal migration (Okubo et al., 2006). In addition, applying protein to rodents lacking the gene have suggested Anosmin-1 regulates cell adhesion (Soussi-Yanicostas et al., 1998; Bribian et al., 2008), migration (Cariboni et al., 2004), and neurite outgrowth and branching (Soussi-Yanicostas et al., 2002; Diaz-Balzac et al., 2015). Mutations in *ANOS1*, account for approximately 10–20% of KS cases. These mutations include frameshift, nonsense mutations, and a few missense mutations and splicing mutations have been identified (Albuisson et al., 2005; Pedersen-White et al., 2008; Goncalves et al., 2017; Maione et al., 2018). Almost all patients with an *ANOS1* mutation suffer anosmia or hyposmia (Quinton et al., 2001; Goncalves et al., 2017; Nie et al., 2017). Synkinesis and renal agenesis are often observed in these patients but do not always co-segregated with the mutation in the pedigree. Hearing loss and vas deferens agenesis are also reported phenotypes occasionally seen in patients with *ANOS1* mutation (Dode and Hardelin, 2010; Madhu et al., 2012; Costa-Barbosa et al., 2013; Xu et al., 2015).

#### FGF17

FGF17 has strong sequence homology, similar splicing, and a similar FGFR1 binding specificity as FGF8 (Olsen et al., 2006; Valdes-Socin et al., 2014). During development, FGF17 expression overlaps with FGF8 in the olfactory placodes, where FGF17 is expressed specifically in the ectoderm at the medial side of the nasal pits from which GnRH cells eventually arise (Bachler and Neubuser, 2001). In addition, FGF17 is important for proper craniofacial development (Kasberg et al., 2013). Although its function is similar to FGF8, FGF17 has a milder phenotype when knocked out in mice. *Fgf17* knockout mice survive into adulthood, display smaller olfactory bulbs but increased volume in the hypothalamus (Yu et al., 2011), and are fertile. In humans, FGF17 mutations have been identified in 2/199 individuals with KS (Miraoui et al., 2013). One of these mutations appeared with other mutations in *FLRT3, HS6ST1, FGFR1*. This same study also proposed that FGF17 served as an alternative ligand to FGF8b in GnRH neuron development, as both FGF8b and FGF17 bind FGFR1c, which is also mutated in some KS individuals.

#### IL17RD

**FIGURE 5:**
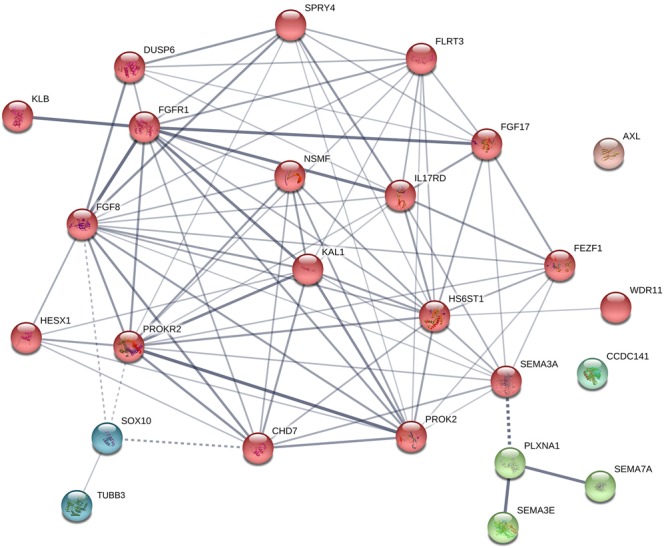
Protein–protein interaction network in known genes of KS. The diagram shows known and predicted interactions among KS causative genes listed in Table 2, created using String software version 11.0 with minimal confidence score of 0.4. Total 87 lines (interactions) appeared across 25 nodes. The colored nodes symbolize proteins which clustered by MCL algorithm. The thickness of the line represents confidence of protein–protein associations (with score 0.4, 0.7, and 0.9, relatively). Solid line, interactions within a cluster; Dotted line, interactions between clusters.

Miraoui and colleagues (2013) used targeted Sanger sequencing to perform a functional candidate-gene approach on a large group of KS/nIHH cohorts (386 IHH probands, 199 KS and 187 nIHH), and identified mutations in genes from the *FGF8* subfamily (*FGF17* and *FGF18*) and their synexpression group (*IL17RD, DUSP6, SPRY2, SPRY4*, and *FLRT3*). They found mutations in five of seven genes screened: *IL17RD, FGF17, DUSP6, SPRY4*, and *FLRT3*. *IL17RD* was first identified as synexpression gene of *Fgf* genes during embryonic development (see Figure 5) and named as *SEF* (Similar Expression to *Fgf* genes). SEF expression is positively regulated by FGF and functions as an antagonist of FGF signaling in zebrafish or *Xenopus laevis* (Furthauer et al., 2002; Tsang et al., 2002). Seven different mutations on *IL17RD* have been identified from 8 patients, and 2 of them have additional mutations, one with *FGFR1* and the other with *KISS1R*. All the patients with a mutation in *IL17RD* were anosmic. 75% of these patients exhibited hearing loss (Miraoui et al., 2013). Some of the patients also exhibited abnormal dentition, or low bone mass. A functional relation of *IL17RD* with the auditory system has been described in two models. Defected auditory development is observed in overexpressed chick and mutant mice (Abraira et al., 2007). These data suggest that mutations in genes encoding components of the FGF pathway are associated with complex modes of KS inheritance and act primarily as contributors to an oligogenic genetic architecture of this disease.

#### DUSP6

Dual specificity phosphatase 6 (*DUSP6*), encodes MKP3, a Mitogen-activated protein kinase (MAPK) phosphatase which inhibits the MAPK pathway, and therefore FGF signaling, via dephosphorylation of active extracellular-signal-regulated kinase (ERK), in negative feedback loop (Li et al., 2007). DUSP3/MKP3 is expressed during embryonic development in areas corresponding to FGF signaling including the forebrain and OE, and requires FGF signaling for its expression (Ishibashi et al., 2001; Kawakami et al., 2003; Li et al., 2007; Urness et al., 2008). The loss of MKP3 is postnatally lethal (Li et al., 2007). In humans, mutations in *DUSP6* have been associated with KS (2.5%). Miraoui et al. (2013) identified 5 KS individuals with *DUSP6* mutations, three of which occurred with other mutation in FGF8 network genes (*SPRY4* and *FGFR1*).

#### SPRY4

Sprouty homolog 4 (SPRY4) is a cytoplasmic membrane-associated protein that inhibits receptor tyrosine kinase signaling and antagonizes FGF, EGF, and VEGF signaling via MAPK pathway inhibition (Hacochen et al., 1998; de Maximy et al., 1999). SPRY4 expression is regulated by FGF signaling (Minowada et al., 1999), and is found in the developing brain, nasal placode, inner ear, limb buds, branchial arches, tail bud, lungs, and areas of the developing gut (de Maximy et al., 1999; Zhang et al., 2001). In mice, the loss of SPRY4 resulted in dwarfism, polysyndactyly (a combination of polydactyly and syndactyly where extra digits are formed but contained in a single skin envelope), some abnormal tooth development, and mandible (jawbone) defects (Taniguchi et al., 2007). Mutations in *SPRY4* were identified in 9 KS patients, and two showed loss of bone mass, three had hearing loss, and one had abnormal dentition. Three of the KS patients also had mutations in additional FGF8 network genes (*DUSP6* and *FGFR1*, Miraoui et al., 2013).

#### *FLRT3* (Fibronectin Leucine-Rich Transmembrane Protein 3)

*FLRT3* is a putative type-I transmembrane protein (Lacy et al., 1999; Haines et al., 2006). FLRT3 expression overlaps with some FGF8 expression and is regulated by FGF signaling (Bottcher et al., 2004). FLRT3 interacts with FGFR1 and promotes FGF signaling via MAPK pathway during early development (Bottcher et al., 2004; Haines et al., 2006). FLRT3 is a cell surface adhesion protein that is critical for neurite outgrowth (Robinson et al., 2004; Tsuji et al., 2004), and interacts with the receptor ROBO1 for Netrin-1 dependent axon guidance (Leyva-Diaz et al., 2014). *Flrt3* knockout mice die at early stages due multiple developmental defects (Egea et al., 2008; Maretto et al., 2008). Miraoui et al. (2013) identified 3 KS individuals (1.5%) with mutations in *FLRT3*. However, 2 of these patients also had mutations in other FGF-network-associated genes, including *FGFR1*.

#### KLB (Klotho Beta)

Xu et al. (2017) examined the FGF21/FGFR1/Klotho Beta (KLB) signaling pathway that mediates the response to starvation and other metabolic stresses in 227 KS patients. KLB and FGFR1 are co-receptors for FGF21. KLB binds to the FGFR1, competing with FGF8 for the same binding pocket (Goetz et al., 2007, 2012; Kurosu et al., 2007; Ogawa et al., 2007; Owen et al., 2015; Misrahi, 2017). Large scale genetic screening revealed six different heterozygous *KLB* mutations in 11 KS patients. Some of these patients had a metabolic phenotype as well, such as obesity and insulin resistance. Mutations occurred as autosomal dominant, *de novo*, or oligogenic in a pedigree. 2/11 of these patients had no other identified mutation. The other patients were found to have mutations in *FGF8* or *PROKR2* or *FGFR1* together with the *KLB* mutation. Double KO of two *KLB* homologs in *C. elegance, klo-1* and *klo-2*, resulted in subfertility, with a 40% reduction of ∼40% egg laying compared to WT (Xu et al., 2017).

#### FEZF1

KO in *Fezf1* was known to alter olfactory axon outgrowth and GnRH neuronal migration (Watanabe et al., 2009) (see Section “Migration of GnRH Neurons From the Nose to the Brain”). Two homozygous mutations in *FEZF1* were identified from two different KS families through the exome sequencing and autozygosity mapping (Kotan et al., 2014). One missense mutation was detected together with a second homozygous mutation in the *CCDC141* (Hutchins et al., 2016; Turan et al., 2017). These patients did not show any additional features associated with KS such as cleft palate, deafness, and synkinesis. The phenotype of the patients suggests that *FEZF1* is spatially and temporally restricted in expression and functionally specific during embryogenesis. The *FEZF1* missense mutation exhibited partial function *in vitro* as a transcriptional repressor suggesting the involvement of the *CCDC141* mutation in the KS phenotype (Kotan et al., 2014).

#### HESX1

A screen of KS patients identified three heterozygous missense *HESX1* mutations (3.6%) (Newbern et al., 2013). HESX1 is a paired-like homeobox transcriptional repressor that is first expressed at the onset of gastrulation. The *Hesx1* homozygous mutant mouse generated two classes of phenotypes. The most severe (class I) consisted of substantial reduction of the prospective forebrain, Rathke’s pouch, olfactory placodes and frontonasal mass along with loss of optic vesicles. Class II was milder with an overall reduction of the forebrain and less craniofacial dysplasia, thinner OE with reduced or absent VNO, abnormal Rathke’s pouch, and usually only one eye affected (Dattani et al., 1998). Because HESX1 is required for pituitary development, the KS patients with mutations in this gene may have low LH/FSH levels related to pituitary defects and/or defects in the development of the GnRH system. To date, a model to examine this issue has not been explored.

#### TUBB3

*TUBB3* is originally identified as a genetic cause of congenital fibrosis of the extraocular muscles (CFEOM), a complex dysmotility disorder characterized by ptosis and strabismus, with or without additional neurological findings. However, detailed phenotypic analysis on patients with *de novo* heterozygous mutation c.1228G > A (p. E410K) showed additional phenotypes such as KS, intellectual disability, facial weakness, tracheomalacia, vocal cord paralysis and late onset cyclic vomiting and progressive peripheral neuropathy. TUBB3, primarily expressed in neurons, is a class III member of the beta-tubulin protein family, which heterodimerizes with alpha-tubulin to form microtubules. *In vitro* functional assays showed all the mutations identified in *TUBB3* altered microtubule dynamics, and some of mutations (including c.1228G > A (p. E410K)) also perturbed interaction of microtubule with kinesin (Tischfield et al., 2010). Chew et al. (2013) suggested defining a distinctive “*TUBB3* E410K syndrome.” All tested patients with E410K showed strong penetrance of the KS phenotype (hypoplasia to absent olfactory sulci, and olfactory bulbs, and hypogonadotropic hypogonadism) without any additional mutation in other candidate genes (Chew et al., 2013). However, in 2015, a female patient with a E410K mutation in *TUBB3* was identified and exhibited normal fertility yet displayed all other features of the E410K syndrome, including anosmia. This heterozygous mutation was inherited by her three sons, as well as variable features of additional endocrine abnormalities (Balasubramanian et al., 2015). *Tubb3* KO mice show no notable neurobehavioral or neuropathological deficits that overlap with above described patient (Latremoliere et al., 2018).

#### *HS6ST1* (Heparan Sulfate 6-*O*-Sulfotransferase 1)

*HS6ST1* is one of three vertebrate genes that encodes an enzyme with HS 6-*O*-sulfotransferase activity (Habuchi et al., 2000). HS6ST1 is strongly expressed in the olfactory nerves, and OE (Nishizuka and Arai, 1996; Sedita et al., 2004). Studies have suggested that anosmin-1 require heparan sulfate with specific 6-*O*-sulfate modifications to exert its function (Bulow et al., 2002; Loo and Salmivirta, 2002; Tornberg et al., 2011). Out of 338 KS/nIHH patients, 5 different heterozygous missense mutations in *HS6ST1* were identified from 7 patients (5 KS patients and 2 nIHH patients) through targeted sanger sequencing (2% of IHH patients). Of these 5 mutations, 2 exhibited digenicity in KS patients, by co-existing with a second heterozygous mutation in *FGFR1* or *NSMF* (Tornberg et al., 2011). Cleft palate occurred in some patients, but not always together with the second mutation, even in a same pedigree revealing variable expression of phenotype. Most *Hs6st1*-KO mice die during embryonic development, between E15.5 and the perinatal stage, but a few mice survive and as adults exhibited erroneous axon navigation in the optic chiasm (Pratt et al., 2006; Habuchi et al., 2007; Izvolsky et al., 2008). The fertility of *Hs6st1* KO mice has not been reported.

#### CHD7

CHD7 is expressed in undifferentiated neuroepithelium and neural crest originated mesenchyme (Sanlaville et al., 2006). Several other genes known to be regulated by *CHD7* are essential for the neural crest transcriptional circuitry, such as *SOX9, TWIST*, and *SLUG* (Bajpai et al., 2010). Because of its early and wide expression during development, mutations in *CHD7* are associated with problems throughout the entire body, and the disorder is categorized as CHARGE syndrome (Coloboma, Heart malformation, Atresia choanae, Retardation of growth and/or development, Genital anomalies, and Ear anomalies/deafness). Since IHH and olfactory defects are common phenotypes in both CHARGE syndrome and KS, KS is considered a part of the CHARGE syndrome spectrum (Pinto et al., 2005; Kim et al., 2008). Two recent studies showed that patients harboring missense mutations in *CHD7* predominantly suffer KS, whereas truncating mutations (nonsense, frameshift, and splicing mutations) mostly result in traditional CHARGE syndrome (Balasubramanian et al., 2014; Marcos et al., 2014). *CHD7* mutations are ∼1.5% of the KS patients (Balasubramanian and Crowley, 2017). Some patients with *CHD7* mutations also possessed mutations in *FGFR1* and *SEMA3A*, supporting oligogenism of KS. Several animal models of *CHD7* has been generated, with the phenotype mimicking that seen in CHARGE patients. Nine ENU (*N*-ethyl-*N*-nitrosourea) induced *Chd7* heterozygous mutant mice exhibited cleft palate, choanal atresia, septal defects of the heart, hemorrhages, prenatal death, vulva and clitoral defects, and keratoconjunctivitis sicca (Bosman et al., 2005). While a study of *Chd7* null mice reported that the loss of a single allele of *Chd7* results in smaller olfactory bulbs and reduced olfactory sensory neurons (Layman et al., 2009).

#### *WDR11* (WD Repeats 11)

WD repeats are minimally conserved regions of approximately 40 amino acids that facilitate the formation of multiprotein complexes (Smith, 2008). They are widely expressed and poorly understood. In E10.5-E14.5 mice, WDR11 is expressed in the developing CNS except the spinal cord. *Wdr11*-null mice exhibit olfactory bulb dysgenesis, hypothalamic GnRH deficiency, pituitary dysgenesis, delayed puberty and reproductive dysfunctions, and obesity (Kim et al., 2018). Notably, intense expression is detected in the diencephalic neuroepithelium and the olfactory bulb neuroepithelium (Kim et al., 2010). Roles of WDR11 protein include regulating the expression of GnRH1 (Kim et al., 2010, 2018). Kim et al. (2010) found a heterozygout missense muation from KS patients which abolishes binding with its partner, EMX1. Only one mutation has been identified in KS while 6 other mutations are founded from nIHH and other HPG axis diseases (combined pituitary hormone deficiency, CPHD, and pituitary stalk interruption syndrome, PSIS) (Izumi et al., 2014; McCormack et al., 2017).

#### AXL

*AXL* encodes a receptor tyrosine kinase which is a member of the TYRO3, AXL, and MER (TAM) family and has been mutated in 1.9% of KS patients (Salian-Mehta et al., 2014). These receptor tyrosine kinases form homodimers or heterodimers with each other and have a multitude of functions in response to ligand binding, i.e., Growth arrest specific gene 6 (GAS6) and protein S (Allen et al., 2002; Pierce et al., 2008). *Axl*^-/-^ and *Tyro3*^-/-^ double KO mice showed a delay of first estrus, persistent abnormal estrus cyclicity, and selective loss of GnRH neurons (36%) in the ventral forebrain, and increased apoptosis along the GnRH migratory route (Pierce et al., 2008). *Axl*^-/-^ only mice exhibited delayed first estrus and an increased interval between vaginal opening and first estrus. However, estrus cyclicity was normal in adult mice, suggesting compensation (Salian-Mehta et al., 2014).

#### CCDC141 (CAMDI)

Not a lot is known about CAMDI, a protein encoded by *CCDC141*. Experiments suggest that it is involved in radial migration, interacting with myosin II and DISC1, and regulates centrosome positioning during neuronal development (Fukuda et al., 2010). CAMDI can also bind to HDA C6 and inhibit the -tubulin deacetylase activity at the centrosome (Fukuda et al., 2016). Five different mutations (one nonsense and four missense) in the *CCDC141* gene have been identified (Turan et al., 2017). Relevant to KS patients, *Ccdc141* was found to be is expressed during development in migrating GnRH neurons, olfactory sensory neurons, nasal mesenchymal cells, (Hutchins et al., 2016). *In vitro* migration assays using siRNA showed knock down of *Ccdc141* resulted in a 25% reduction in migration rates of GnRH neurons, but olfactory axon growth was not affected (Hutchins et al., 2016). Using *Ccdc141* KO mice, Fukuda et al. (2016) reported delayed radial cell migration, abnormal neural circuit formation, and psychiatric behaviors, but that the mice were fertile. Based on the analysis of *Ccdc141* splice variants in mice and humans (unpublished data obtained from open access databases) it is possible that the KO mouse used by Fukuda et al. (2016) still produces a *Ccdc141* isoform(s) that may compensate in the GnRH system. To unveil the role of CCDC141 involvement with pathogenic mechanism of KS, a complete null model of *Ccdc141* or mutation knock-in model will be required.

## Bioinformatic Analysis on Ks Genes

**FIGURE 6:**
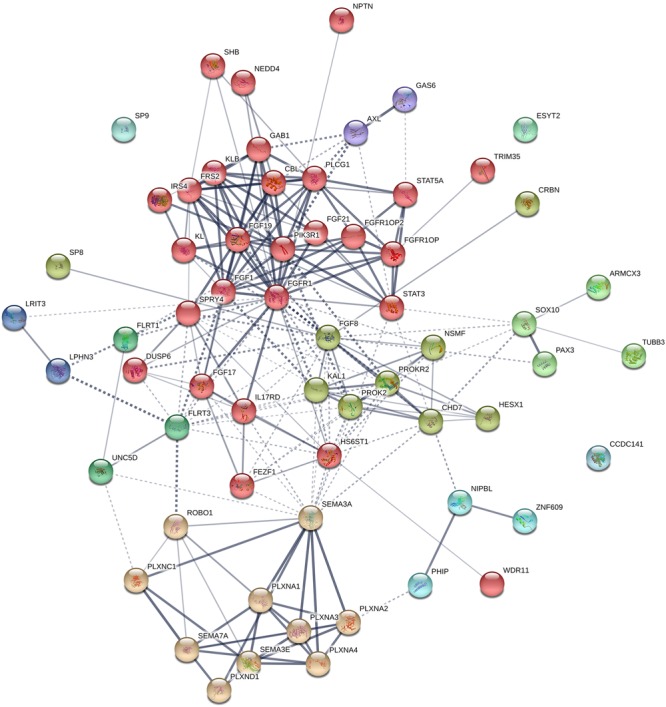
Protein–protein interaction network in known genes of KS and their interacting partners. The diagram shows known and predicted interactions among KS causative genes and their interacting proteins, created using String software version 11.0 with minimal confidence score of 0.4. Total 227 lines (interactions) appear across 63 nodes (25 known factors of KS and 38 additional interacting proteins). The colored nodes symbolized proteins which clustered by MCL algorithm. The thickness of the line represents confidence of protein–protein associations. Solid line, interactions within a cluster; Dotted line, interactions between clusters. Abbreviations for 38 proteins interacting with known KS factors: ARMCX3, Armadillo repeat-containing X-linked protein 3; CBL, E3 ubiquitin-protein ligase CBL; CRBN, Protein cereblon; ESYT2, Extended synaptotagmin-2; FGF1, Fibroblast growth factor 1; FGF19, Fibroblast growth factor 19; FGF21, Fibroblast growth factor 21; FGFR1OP, FGFR1 oncogene partner; FGFR1OP2, FGFR1 oncogene partner 2; FLRT1, Leucine-rich repeat transmembrane protein FLRT1; FRS2, Fibroblast growth factor receptor substrate 2; GAB1, GRB2-associated-binding protein 1; GAS6, Growth arrest-specific protein 6; IRS4, Insulin receptor substrate 4; KL, Klotho; LPHN3, Adhesion G protein-coupled receptor L3; LRIT3, Leucine-rich repeat; NEDD4, E3 ubiquitin-protein ligase NEDD4; NIPBL, Nipped-B-like protein; NPTN, Neuroplastin; PAX3, Paired box protein Pax-3; PHIP, PH-interacting protein; PIK3R1, Phosphatidylinositol 3-kinase regulatory subunit alpha; PLCG1, 1-phosphatidylinositol 4,5-bisphosphate phosphodiesterase gamma-1; PLXNA2, Plexin-A2; PLXNA3, Plexin-A3; PLXNA4, Plexin-A4; PLXNC1, Plexin-C1; PLXND1, Plexin-D1; ROBO1, Roundabout homolog 1; SHB, SH2 domain-containing adapter protein B; SP8, Transcription factor Sp8; SP9, Transcription factor Sp9; STAT3, Signal transducer and activator of transcription 3; STAT5A, Signal transducer and activator of transcription 5A; TRIM35, Tripartite motif-containing protein 35; UNC5D, Netrin receptor UNC5D; ZNF609, Zinc finger protein 609.

A protein–protein interaction network analysis of known genes of KS was performed to identify new candidates that may be involved in KS. The interactions across these 25 proteins were determined by STRING analysis^1^ (Figure 5). Most of proteins interact with each other, and can be clustered into three different groups, except CCDC141 and AXL. The main cluster (red colored) shows robust interactions among 18 proteins. The Semaphorins and their receptors (green colored) are linked to the main cluster through SEMA3A. TUBB3 and SOX10 (dark cyan colored) are linked to main cluster as well, through SOX10 and its interacting partners (FGF8, PROKR2, and CHD7). Based on the STRING database, 38 additional proteins that interact with known factors of KS were predicted (Figure 6). The interactions through these genes indicate that they might have a role in the development of the olfactory/GnRH systems. As such, these proteins are potential candidate genes for genetic screening of KS patients who don’t harbor mutations in any of the known genes associated with KS.

Some of the genes involved in KS/nIHH have been studied to investigate their pathogenic mechanism in the disease and to get a better understating of their overall role in olfactory development and GnRH neuronal migration. However, many of the genes, at least those associated with KS, are broadly expressed during development rather than restricted to the nasal area. This makes it difficult to focus on the molecular function of these gene products at the cellular level, in the cell of interest. To begin to address this issue, we compared the expression of known causative genes of KS/nIHH in three different cell types involved in olfactory/GnRH development and function; OSNs, OECs and GnRH cells (Table 2). The expression levels are represented by FPKM (Fragments Per Kilobase Million) values were gathered from Gene Expression Omnibus (GEO) repository^2^ and other studies including (1) OSN data set (GSE53793) – collected from 4 weeks old OMP-GFP mice (6 homozygous and 8 heterozygous) by FACS of OMP-GFP positive cells and following RNA-Seq analysis (Kanageswaran et al., 2015), (2) OEC data set (GSE69312) collected from cultured human immortalized OEC cells and following RNA-Seq analysis (Dando et al., 2016), unfortunately such data from mice is currently not available) and (3) GnRH data set collected from adult GnRH-Cre/L10a-GFP mice by immunoprecipitation of GnRH specific GFP positive ribosome-mRNAs complex (polysomes) and following RNA-Seq analysis (Burger et al., 2018).

*TUBB3, NSMF*, and *STUB1* are abundantly expressed in all of three cell types (18.119 ≤ FPKM ≤ 666.014). *SOX2, SOX10, STUB1, GNRH1, KISS1*, and *KISS1R* are highly expressed in GnRH cells (12.002 ≤ FPKM ≤ 37010.3). The role of SOX2 and SOX10 in GnRH cells is unclear. Certainly, the expression of *GNRH1, KISS1*, and *KISS1R* correlates with their role in GnRH neuronal function. *IL17RD, FEZF1, HS6ST1, CHD7, WDR11, SEMA3E, SEMA7A, PLXNA1, OTUD4, RNF216, POLR3A*, and *DMXL2* showed the highest expression in OSNs among three cell types. The expression levels of *FGFR1, DUSP6, SPRY4, AXL, PNPLA6*, and *LEPR* are higher in OECs compare to other cell types. *HS6ST1, WDR11, SEMA7A, RNF216*, and *PNPLA6* are relatively high in both OSNs and OECs. The differential expression of these genes may indicate the primary cell that is perturbed and underlies the KS phenotype. Many of genes related with nIHH (*GNRHR, TAC3, TACR3, LEP, NR0B1, FSHB, LHB, LHX4, PROP1*, and *PCSK1*) showed low expression (FPKM ≤ 3.016) in these 3 cell types suggesting an indirect role in either GnRH development or physiology.

**FIGURE 7:**
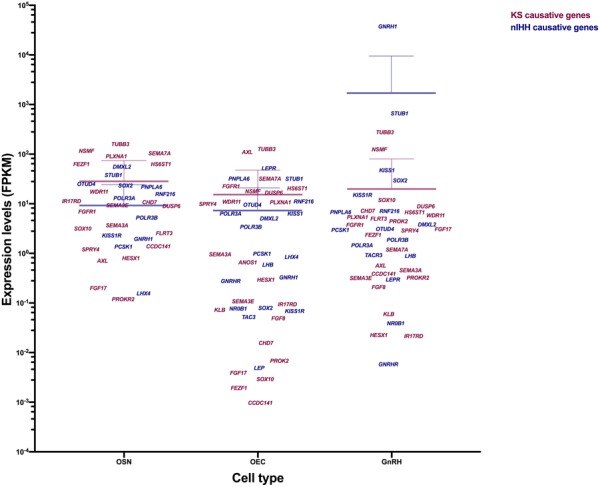
Expression level of genes causing KS vs. nIHH in OSN, OEC, and GnRH neurons. Genes related to KS (magenta) and genes only related to nIHH (blue) are plotted according to their expression level (FPKM) in the three different cell types. The gene expression values are gathered from previous studies and GEO database and compared in OSN (GSE53793), OEC (GSE69312) and GnRH neuron (Burger et al., 2018). The mean expression level and SD of gene group is represented as same color on the scattered plot.

The mean expression values of the genes related to olfactory symptoms (KS, magenta) are higher than the other genes (only identified in nIHH patients, blue) in OSN (KS, mean 28.36 ± SD 45.89, nIHH, mean 9.34 ± SD 15.15) and OECs (KS, mean 15.28 ± SD 32.54, nIHH, mean 7.37 ± SD 13.72) while the gene set identified in nIHH patients has a higher mean expression than KS genes in GnRH cells (KS, mean 19.79 ± SD 60.43, nIHH, mean 1718 ± SD 7884) (Figure 7). This is consistent with the KS phenotype and the role of OSNs and OECs in olfactory/GnRH development and GnRH cell migration. In addition, the genes showing high expression in the GnRH cells may be more important for GnRH cell maintanence and survival, and/or neuroendocrine function, which links these genes to the pathogenicity of IHH without olfactory problems. These results suggest that examining gene expression levels in each cell type may uncover the role of these genes in KS vs. nIHH.

## Summary

Kallmann Syndrome is a rare disorder which shows high genetic heterogenicity and oligogenicity. We expect that the results from *in silico* analyses of known factors of KS, together with the updated knowledge on GnRH neuronal development/migration summarized in this review, provides fundamental data that (1) can be used to identify the remaining 60% of the genetic basis of KS, (2) provides new insight into the molecules/mechanisms by which neurons migrate to their appropriate location and our understanding of neuronal disorders associated with aberrant cell migration.

## Data Availability

All datasets generated for this study are included in the manuscript and/or the supplementary files.

## Author Contributions

NW contributed to section “Development of the Olfactory Placode and GnRH Neurons.” YS contributed to section “Migration of GnRH Neurons From the Nose to the Brain.” H-JC contributed to sections “Spectrum of Genetic Disorders in GnRH Cell Deficiency and Kallmann Syndrome” and “Bioinformatic Analysis on KS Genes.” SW supervised the review. All authors wrote and reviewed the manuscript.

## Conflict of Interest Statement

The authors declare that the research was conducted in the absence of any commercial or financial relationships that could be construed as a potential conflict of interest.

## Footnotes

1 http://www.string-db.org

2 https://www.ncbi.nlm.nih.gov/geo
